# Supplementary material for: Mutations in PCYT2 disrupt etherlipid biosynthesis and cause a complex hereditary spastic paraplegia
Source: Brain. 2019 Oct 22;142(11):3382–97. doi: 10.1093/brain/awz291 (PMC6821184; doi:10.1093/brain/awz291)
Supplement: awz291_Supplementary_Data [file awz291_supplementary_data.zip › awz291-Suppl_data/Supplementary_Data4.zip!/Supplementary information 4 - plasma lipidomics.zip!/Plasma lipidomics/Control vs Patient/Statistical plots and data/SM[d] species/Volcano plot_alternative.pdf]

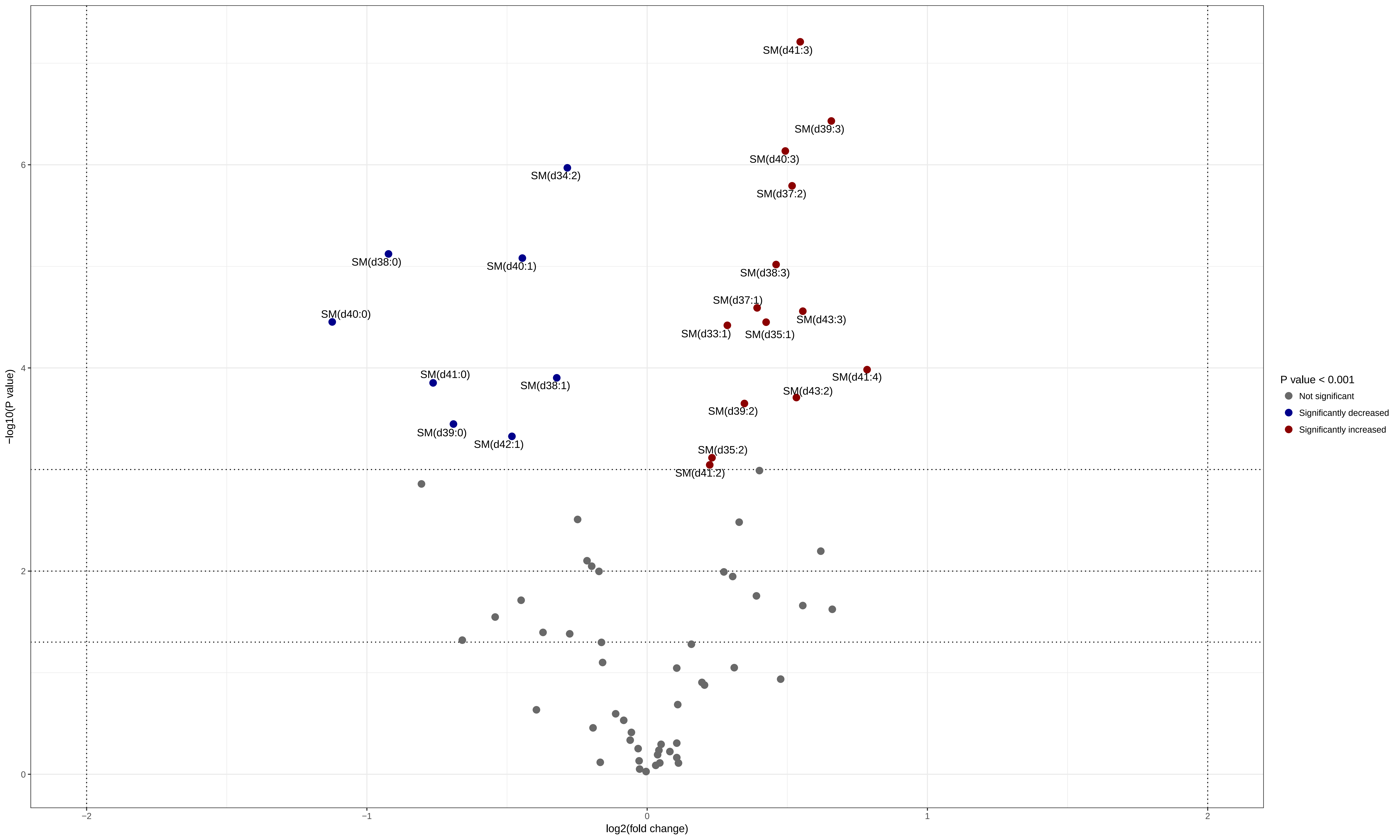
SM(d41:3)
SM(d39:3)
SM(d40:3)
SM(d34:2)
SM(d37:2)
SM(d38:0)
SM(d40:1)
SM(d38:3)
SM(d37:1)
SM(d40:0)
SM(d43:3)
SM(d33:1)
SM(d35:1)
SM(d41:0)
SM(d38:1)
SM(d41:4)
SM(d43:2)
SM(d39:2)
SM(d39:0)
SM(d42:1)
SM(d35:2)
SM(d41:2)
−log10(P value)
log2(fold change)
6
4
2
0
−2
−1
0
1
2
P value < 0.001
Not significant
Significantly decreased
Significantly increased
